# Sketching the landscape: a scoping review of partnerships at the intersection of faith and health

**DOI:** 10.1186/s12889-025-25346-9

**Published:** 2025-12-19

**Authors:** Elizabeth Boutros, Stephanie Beecroft, Sabrina Gupta, Catherine Homer, Mark Cobb, Bruce Rumbold

**Affiliations:** 1 School of Sport and Physical Activity, Sheffield Hallam University, Olympic Legacy Park, 2 Old Hall Road, Sheffield, S9 3TU UK; 2 La Trobe University, Plenty Rd, Bundoora, VIC 3086 Australia; 3 Sheffield Teaching Hospitals NHS Foundation Trust, Sheffield, UK

**Keywords:** Partnership, Collaboration, Faith, Religion, Health, Public health, Health promotion

## Abstract

**Background:**

Much consideration has been given to community-level partnerships in public health. Faith communities are important systems of connection and support for many people but may be overlooked as public health partners in Australia and the United Kingdom. Efforts to enhance community health through partnerships between faith communities and health and wellbeing professionals and organisations have been explored in recent academic literature. Future faith-health partnerships could be enhanced through an examination of the key challenges and facilitators discussed in these articles. This review examined recent literature to determine what health issues were being addressed through partnerships with faith communities, what terminology was used in the literature, where the research was conducted, and what gaps exist in the research.

**Methods:**

Online databases were searched to identify literature published between 2018 and October 2024. Of these, 45 articles were selected for their relevance to the scoping review aims.

**Results:**

Faith-health partnerships implemented health interventions for a range of health issues and populations. Most academic literature from countries culturally similar to Australia and the UK featured research from the USA. Partnerships tended to be described rather than defined, and a range of barriers and facilitators featured in the research.

**Conclusions:**

The facilitators identified could be useful to those wanting to establish faith-health partnerships, but more research is needed into how these challenges and facilitators function. Additionally, research is needed to understand how faith-health partnerships operate in countries outside the USA.

**Supplementary Information:**

The online version contains supplementary material available at 10.1186/s12889-025-25346-9.

## Introduction

Health and wellbeing organisations apply a range of strategies at different levels to improve individual and population health. Partnerships with community groups are routinely used as a strategy to promote health and wellbeing at the community level [1–3]. This has sometimes involved partnering with faith communities [4, 5]. This faith-based approach fits with recommendations from the World Health Organization [6] to strengthen partnerships for health across society.

This scoping review was conducted as part of a realist-informed qualitative research project exploring formal and informal faith-health partnerships in Melbourne, Australia and Sheffield, England. The provision of health and wellbeing services in both countries was historically deeply rooted in faith communities, especially churches, but management and funding were removed from church jurisdiction and taken up by government [7]. Partnerships with faith communities could help address health disparities, particularly in ethnic communities [8], but there is a need to know more about how these partnerships work.

An initial search of the academic literature suggested that limited research has been conducted on faith-health partnerships in Australia and England. Some recent literature has explored the intersection of health and faith responses to public health issues. Idler and Kellehear [9] described different roles that faith institutions play in the health care sectors in the United States of America (USA) and United Kingdom (UK). However, their work focuses specifically on the health care sector, and partnerships with faith communities may take place beyond the boundaries of formal health care systems. Song et al. [10] and Perez et al. [11] have both recently published scoping reviews on partnerships between faith-based organisations or communities, and health programs, focusing on vaccine uptake and mental health respectively. These reviews have made valuable contributions towards understanding how faith-health partnerships have responded to those areas of need, but it would be beneficial to understand the depths and breath of the academic literature examining partnerships with faith communities addressing a broad range of health and wellbeing needs. This could also help to identify what gaps exist in the literature.

Given the heterogeneous nature of the articles found, a formal scoping review of the literature was undertaken to map the academic literature exploring these partnerships, where these studies were taking place, and how partnerships were described [12]. As governments and health organisations look towards improving health through partnership with local communities understanding what is known from recent literature, and where it would be beneficial to focus future research, can help improve evidence-based, collaborative efforts to build healthy communities.

### Rationale

A scoping review was chosen so that common concepts across the literature pertaining to faith-health partnerships could be explored across a heterogenous range of academic literature [13]. The findings were used to inform the research project exploring faith-health collaborations in Melbourne, Australia, and Sheffield, United Kingdom. The PRISMA guidelines for Scoping Reviews was used to structure the reporting of this scoping review [12].

### Objectives

The research question for the scoping review was: What is known from the current literature about the partnerships between faith communities and health and wellbeing advocates who work together to improve the health and wellbeing of the local communities?

For the purpose of this review, faith-health collaborations are defined as formal or informal partnerships between faith communities (for example, places of worship, religious leaders or local groups with self-identified shared religious affiliation) and health or wellbeing advocates (for example, public health professionals or organisations, government departments, clinicians, allied health professionals, community health or development professionals, or health- or wellbeing-focused faith-affiliated organisations or charities). Faith-health collaborations of this kind jointly provide activities to improve health or wellbeing. Activities may include, but are not limited to, education, health screening, health and wellbeing service delivery, advocacy, disease prevention, health promotion, or policy engagement. However, it excludes the involvement of faith communities solely as participants in research or recipients of services or programs, without an active role in collaboration or partnership.

The objectives for the review were to determine what gaps exist in the academic literature by examining (1) what kind of research was carried out, (2) where these studies were conducted, (3) the terminology used in the literature, and (4) how collaboration was conceptualised in the included articles.

Population, Concept and Context (PCC) [14] was used to define the review concepts. These were as follows:

- Population: (1) Faith communities from all world religions, (2) Health and wellbeing organisations and/or professionals (public health, health promotion, health professionals, faith-affiliated organisations, charities).
- Concept: (1) Partnerships, collaborations, or working relationships, being used to (2) address health and wellbeing needs.
- Context: (1) Local community or within the community of faith (2) in United States of America, Canada, United Kingdom, Western Europe, Australia and New Zealand. These countries were chosen after discussion amongst the research team due to their cultural and social similarity to Australia and England, their status as High Income countries or regions, and similarity in health care systems and approaches; thus the most contextually relevant to faith-health partnerships in Australia and England.

## Methods

### Protocol

The review protocol was developed by the researcher (EB) with the assistance of the research team (SB, SG, CH and BR) and a university librarian. The review protocol involved using the Population Concept Context [14] framework to develop inclusion and exclusion criteria for relevant literature. The review protocol development was iterative, as is common in scoping reviews [13]. Inclusion and exclusion criteria were modified over the course of the review to meet the conceptual aim of the research project and to ensure feasibility of the review due to resource and time constraints.

### Eligibility criteria

Eligible articles needed to (1) use the language of partnership or collaboration to describe the relationship between (2) faith community(ies) and health/wellbeing organisations or professionals that were (3) addressing health or wellbeing needs in the selected countries. Most study designs, aside from those listed in the exclusion criteria, were eligible for inclusion in the scoping review, along with theoretical articles published in peer reviewed journals.

Excluded were: (1) articles that did not involve collaboration, such as articles on faith-based health promotion that did not involve external partners, or where faith communities were involved only for research recruitment or as participants in studies; (2) articles focused on projects that were not local to the faith community or health and wellbeing partner, such as international aid projects, or interventions that took place in healthcare settings such as hospitals. Following title and abstract screening, the review team excluded articles which (3) used Community Based Participatory Research (CBPR) methodology. CBPR is an approach to research partnerships underpinned by principles of equality in community participation and contribution [15, 16]. These articles were excluded on the grounds that the research the scoping review was conducted to inform concerned faith-health partnerships which may not involve academic partners and therefore may not involve participatory research approaches such as CBPR. (4) Non peer reviewed material, such as newspaper articles and newsletters, was also excluded, as were dissertations, conference papers, editorials and study protocols.

### Information sources

**Fig. 1:**
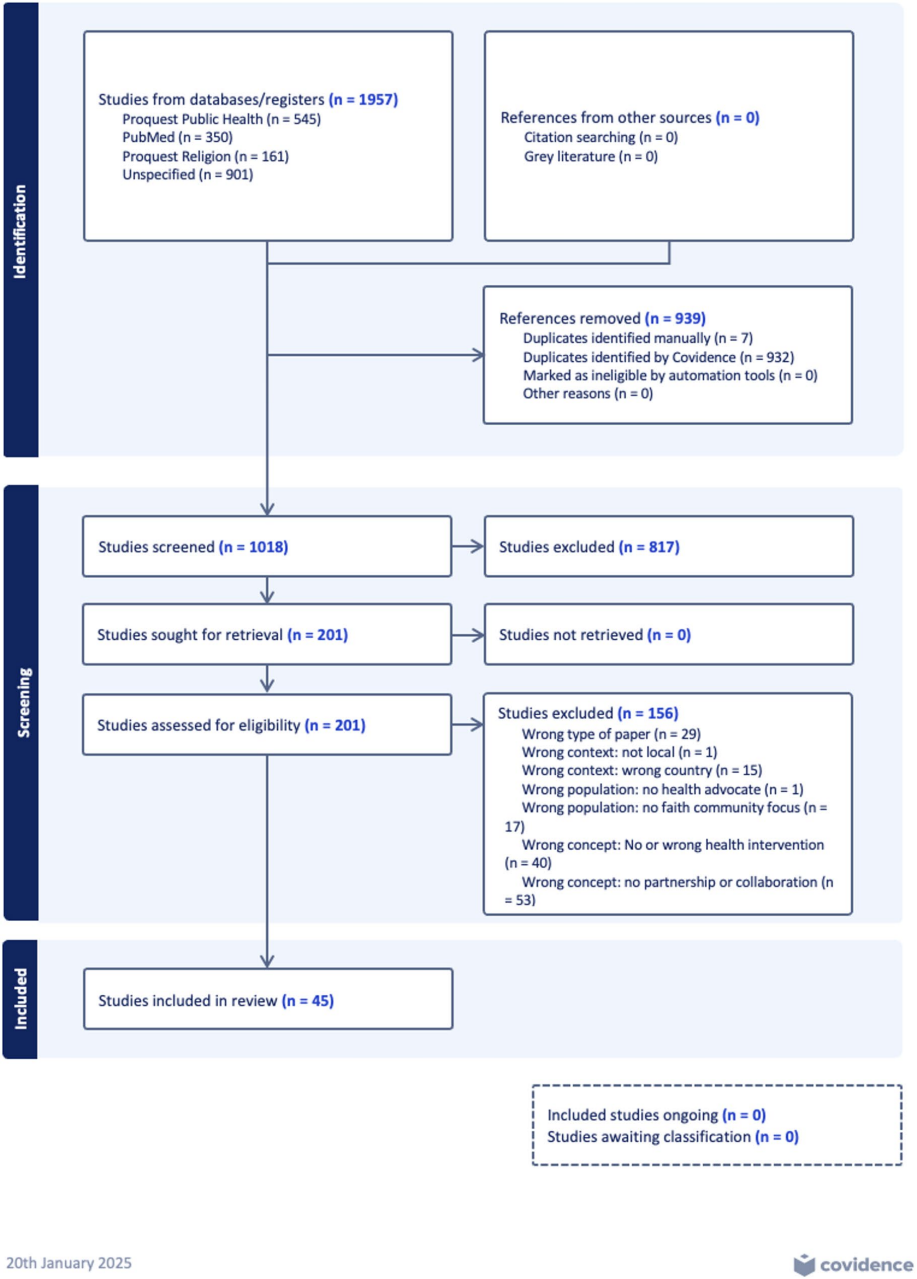
PRISMA Diagram of included and excluded articles

Proquest Public Health, Proquest Religion, and PubMed databases were searched for articles published between the beginning of 2018 and 8th October 2024 that met the inclusion criteria. 2018 was chosen as the point of departure for the research, as it ensured recency of data, and allowed for a feasible number of articles to be screened. It also allowed for some pre-COVID-19 pandemic partnerships to be reviewed for relevant data, as the pandemic constituted a significant shift in the context for public health partnerships. The search strategies were drafted by EB and reviewed by a research librarian. The final search strategy for the above databases can be found in Table 1. The initial search was conducted in March 2024, and an updated search was performed in October 2024. Covidence [17] was used to screen 1957 articles with 932 duplicates removed. An additional four duplicates were manually removed by reviewers (See PRISMA diagram) (Fig. 1).

**Table 1:** Databases, search terms, and search strategy used

Database	Search terms	Search strategy	Filters
Proquest Religion and Proquest Public Health	**Faith-related terms:** Faith community(s) or congregation(s); religious community(s) or congregation(s); Christian community(s), Muslim community(s), Sikh community(s), Jewish community(s), Hindu community(s), Buddhist community(s), parish(es), church(es), mosque(s), masjid(s), jamaat(s), gurdwara(s), temple(s), synagogue(s), mandir(s) **Partnership-related terms:** Health, well(-)being, public health, health promoting, health promotion	((noft(Faith) NEAR/3 noft(communit*)) OR (noft(Religio*) NEAR/3 noft(communit*)) OR (noft(Religio*) NEAR/3 noft(congregation*)) OR (noft(Faith) NEAR/3 noft(congregation*)) OR noft(Parish*) OR noft(Church*) OR noft(Mosque*) OR noft(Masjid*) OR noft(Jamaat*) OR noft(gurdwara*) OR noft(temple*) OR noft(synagogue*) OR noft(Mandir*) OR (noft(Christian*) NEAR/3 noft(communit*)) OR (noft(Muslim*) NEAR/3 noft(communit*)) OR (noft(Sikh*) NEAR/3 noft(communit*)) OR (noft(Jew*) NEAR/3 noft(communit*)) OR (noft(Hindu*) NEAR/3 noft(communit*)) OR (noft(Buddhis*) NEAR/3 noft(communit*))) AND (noft(collaborat*) OR noft(partner*)) AND (noft(health) OR noft(well?being) OR noft("public health") OR (noft("health promoting") OR noft("health promotion")))	Since 2018
Pubmed	**Faith-related terms:** Faith community(s) or congregation(s); religious community(s) or congregation(s); Christian community(s), Muslim community(s), Sikh community(s), Jewish community(s), Hindu community(s), Buddhist community(s), parish(es), church(es), mosque(s), masjid(s), jamaat(s), gurdwara(s), temple(s), synagogue(s), mandir(s) **Partnership-related terms:** Health, well(-)being, public health, health promoting, health promotion	(((((((((((((((((((((((((((((((("Religious community"[Title/Abstract]) OR ("Religious communities"[Title/Abstract])) OR ("Faith community"[Title/Abstract])) OR ("Faith communities"[Title/Abstract])) OR ("Faith congregations"[Title/Abstract])) OR ("Faith congregation"[Title/Abstract])) OR ("Religious congregation"[Title/Abstract])) OR ("Religious congregations"[Title/Abstract])) OR (Parish*[Title/Abstract])) OR (Church*[Title/Abstract])) OR (Mosque*[Title/Abstract])) OR (Masjid*[Title/Abstract])) OR (Jamaat*[Title/Abstract])) OR (Gurdwara*[Title/Abstract])) OR (Temple*[Title/Abstract])) OR (Synagogue*[Title/Abstract])) OR (Mandir*[Title/Abstract])) OR ("Christian community"[Title/Abstract])) OR ("Christian communities"[Title/Abstract])) OR ("Muslim community"[Title/Abstract])) OR ("Muslim communities"[Title/Abstract])) OR ("Islamic community"[Title/Abstract])) OR ("Islamic communities"[Title/Abstract])) OR ("Sikh community"[Title/Abstract])) OR ("Sikh communities"[Title/Abstract])) OR ("Jewish community"[Title/Abstract])) OR ("Jewish communities"[Title/Abstract])) OR ("Hindu community"[Title/Abstract])) OR ("Hindu communities"[Title/Abstract])) OR ("Buddhist community"[Title/Abstract])) OR ("Buddhist communities"[Title/Abstract]) AND (y_5[Filter])) AND ((Collaborat*[Title/Abstract]) OR (Partner*[Title/Abstract]) AND (y_5[Filter]))) AND ((((((health) OR (wellbeing)) OR (well-being)) OR ("well being")) OR ("public health")) OR ("health promot*") AND (y_5[Filter]))	Last 5 years

### Search strategy

Search terms included *Faith-related terms***:** faith community(s) or congregation(s); religious community(s) or congregation(s); Christian community(s), Muslim community(s), Sikh community(s), Jewish community(s), Hindu community(s), Buddhist community(s), parish(es), church(es), mosque(s), masjid(s), jamaat(s), gurdwara(s), temple(s), synagogue(s), mandir(s); as well as *Partnership-related terms:* health, well(-)being, public health, health promoting, health promotion. The search strategies for each database are outlined in Table 1.

### Selection of sources of evidence

Title and abstract screening were conducted by EB, SG and CH, with the assistance of a research assistant. Inclusion and exclusion criteria were refined after title and abstract screening. A flow chart was developed by EB to help with determining what articles should be included or excluded during full text review, based on the PCC criteria (Additional File 1). Full text screening and data extraction were completed by EB and SB. Conflicts were resolved during both title and abstract, and full text screening through discussion between reviewers. Each reviewer presented their rationale for the inclusion or exclusion of an article and discussed until consensus was achieved.

### Data charting process

A data extraction form was created in Covidence by EB based on the objectives of the review (Additional File 2), which was reviewed through discussion with co-authors, and piloted with SB on three articles before data extraction commenced. During and following data extraction, EB and SB discussed rationale for extracting data where disagreements occurred; these were discussed until consensus was reached about the final data to be included in the review. An exception was made for data about the aims of the articles, and how the articles described partnership, where data provided by both reviewers was included. As the extracted data were qualitative in nature and concerned with the description of collaboration, extractor discretion and interpretation were required. Including data from both extractors allowed for a greater breadth in data extraction and for differences in extractor interpretation to be integrated into the analysis.

EB exported Covidence data into Microsoft Excel for cleaning, Microsoft Word for arrangement into tables to enable frequency counts and readability of data, and NVivo [18] for qualitative analysis of data about the aim of articles, purpose of partnerships, facilitators and challenges. Saldana’s [19] two cycles of coding were used. First cycle coding involved assigning codes to segments of text based on semantic, surface-level meaning. Second cycle coding involved consolidating the codes around shared meaning (Table 2).

**Table 2:** Qualitative codes, descriptions, sources, and illustrative quotes of extracted data

**Codes **	**Description**	**Sources *(n=)***	**Illustrative quotes**
*Category: article aims*			
Aims of article – collaboration focused	Aims of an article were collaboration-focused when they were concerned with description or discussing existing or theoretical partnership or collaboration. These included descriptions and evaluation of partnerships, descriptions of partnership models, and theoretical explanations of partnerships.	Theoretical articles: [20–25]. *(n=6)*	“describe the collaborative effort” (Bradley et al, 2018, p.730) [26] “we aim to explore how these collaborative negotiations were established among the Montreal Regional Public Health Unit (PHU), a transcultural psychiatry team (TP), the police, and two different religious communities and to identify the outcomes, obstacles and facilitators.” (El-Majzoub et al., 2021, p.4566) [27]
		Empirical articles: [28–32]. *(n=5)*	
		Program description articles: [27, 33–36]. *(n=5)*	
		Program evaluation articles: [26, 37, 38]. *(n=3) *	
Aims of article – intervention focused	Aims of an article were intervention-focused when they described or reported on measurements of a health and wellbeing intervention. This included describing programs, describing intervention developments, describing key learnings from program implementations, determining acceptability of interventions, examine context factors influencing intervention, identifying needs to be targeted by intervention, intervention evaluations, describing pilot interventions,	Theoretical articles: [25, 39, 40]. *(n=3)*	“This article evaluates the utility of commonly used health communication theories for communicating health information about addiction in religious settings and identifies their shortcomings.” (Clements et al., 2021, p.1). [40] “This report describes the NOML program and describes morbid characteristics of NOML attendees at risk for adverse asthma outcomes.” (Harris et al., 2020, p. 624) [41].
		Empirical articles: [32, 41–54]. *(n=15)*	
		Program description articles: [34, 55–58]. *(n=5)*	
		Program evaluation articles: [26, 37, 59–63]. *(n=7) *	
*Category: challenges*			
Challenges – intervention related	Intervention-related challenges involved the identification of factors that prevented interventions from working as well as they could. Examples included challenges with recruitment, readiness, funding, etc.	Theoretical articles: [25]. *(n=1)*	“Faithful Families has experienced significant barriers around data collection, program fidelity, and readiness.” (Hardison-Moody & Yao, 2019, p.365) [57]
		Empirical articles: [45, 47]. *(n=2)*	
		Program descriptions articles: [57]. *(n=1)*	
		Program evaluations articles: [26]. *(n=1)*	
Challenges - relational	Relational challenges involved the identification of factors that affected relationships. Examples included apathy, distrust and navigating differing priorities and values.	Theoretical articles: [24]. *(n=1)*	“I outline some of the key ethical issues that are encountered in community clinics, and our clinic specifically, including how to […] balance different values and priorities within the partnership.” (Moore, 2024, p.209) [35]. “we found that churches were eager to partner with the RADx-UP initiative to combat COVID-19 vaccine hesitancy but were sometimes met with negative responses and apathy from their congregations and surrounding community.” (Bateman et al., 2024, S3940.) [56]
		Empirical articles: [46, 47]. *(n=2)*	
		Program description articles: [27, 35, 56]. *(n=3)*	
*Category: facilitators*			
Facilitators – asset related	Asset-related facilitators increase program or partnership effectiveness through assets like people, facilities, and resources. Examples include recognising the expertise of each partner, organisational capacity, community reach and reputation.	Theoretical articles: [21–23, 25, 39]. *(n=5)*	“Early in the development of the TPN partnership, we learned that there was great knowledge to be gleaned from our partners, and that this information was as valuable as the health information we intended to share.” (Gwathmey et al., 2024, p.564). [33].
		Empirical articles: [28–32, 41, 45–51, 54, 64]. *(n=15)*	
		Program description articles: [27, 33, 34, 36, 55–57]. *(n=7)*	
		Program evaluation articles: [26, 38, 58–60, 62]. *(n=6) *	
Facilitators – relational	Relational facilitators increase program or partnership effectiveness through relational assets like trust, “bridge building”, a shared sense of values, ideology, or goals, and building partnerships from pre-existing relationships.	Theoretical articles: [21, 25, 39, 40]. *(n=4)*	“The Community Connector role in building rapport and trust, as well as addressing other needed resources, is key to this success.” (Scribner et al., 2020, p.1953). [58] “Healthcare professional experience was described as a strength for those working in church health ministries because it allowed participants to engage in work and build partnerships.” (Fuller et al., 2024). [31]
		Empirical articles: [28–32, 42, 45, 47–49, 51–53]. *(n=13)*	
		Program description articles: [33–36, 55, 57, 58]. (*n*=7)	
		Program evaluation articles: [26, 37, 59, 62, 63].*(n=5)*	
*Category: faith community involvement in public health*			
	Faith communities have characteristics that can facilitate involvement in public health. These include being trust by their communities, being message communicators, provide environments supportive of health, and by increasing access to interventions.	Theoretical articles: *(n=0)*	“Hatzola hosted these vaccination sessions and had responsibility for promotion, distributing appointments to callers and administering vaccines. Events were also supervised by Jewish healthcare professionals working in the community, which offered continuity between delivery of routine vaccinations and the CVP.” (Kasstan et al., 2022, p.2228) [45] “This initiative was developed to connect faith leaders, religious institutions, and community members with the goal of establishing the Black Church as a change agent to overcome stigma through faith leader trainings, and the integration of HIV messages into church activities.” (Bradley et al., 2018, p.732) [26]
		Empirical articles: [32, 45–48, 51, 53]. *(n=7)*	
		Program description articles: *(n=0)*	
		Program evaluations articles: [38, 62, 63]. *(n=3)*	
*Category: models *			
Models – intervention-related	Intervention-related related to the underlying health need were discussed, or existing health promotion models of health promotion were applied, evaluated or adapted in interventions.	Theoretical articles: [21, 39, 40]. *(n=3)*	“The current study evaluated the implicit and explicit impacts of a church-based counseling model known as Church Therapy.” (Kansiewicz & Smith, 2021, p.67) [43]
		Empirical articles: [43]. *(n=1)*	
		Program description articles: [57]. *(n=1)*	
		Program evaluation articles: [26] *(n=1)*	
Models – partnership-related	Partnership-related models underpinned approaches to collaboration.	Theoretical articles: *(n=0)*	“Our approach to creating a successful community partnership employs each of the core principles of community engagement.” (Gwathmey et al., 2024, p.562). [33]
		Empirical articles: [28]. *(n=1)*	
		Program description articles: [33]. *(n=1)*	
		Program evaluation articles: *(n=0)*	
Category: benefit of partnership			
Benefits – intervention-related	Intervention-related benefits included the ability to reach beyond congregations into wider community, increased trust in intervention, and building organisational reputation through being seen to be providing interventions.	Theoretical articles: *(n=0)*	The "optics" of charity care matter” (Moore, 2024, p.212) [35].
		Empirical articles: [32, 53]. *(n=2)*	
		Program description articles: [36]. *(n=1)*	
		Program evaluation articles: *(n=0)*	
Benefits – health-related	Health related benefits included	Theoretical articles: *(n=0)*	“intangible outputs included building social capital and trust in the community, advancing community unity, and increasing individual self-efficacy.” (Mattingly et al. 2024) [28]
		Empirical articles: [28, 44]. *(n=2)*	
		Program description articles: *(n=0) *	
		Program evaluation articles: *(n=0)*	
Benefits – partnership-related	Partnership-related benefitss were about successful collaboration, and may be linked to health-related benefits, as well as recognition of the contribution of faith communities to public health. Partnerships could result in increased sustainability of interventions. However, partnership may lead to criticism of faith community if participants distrust intervention.	Theoretical articles: *(n=0)*	“successful local and regional cooperation is both achievable and, given the right resources and support, highly effective. Taken together, there is strong evidence here to suggest that a more collaborative approach will improve mental health services and outcomes for Muslim communities in England and Wales.” (Abrar & Hargreaves, 2024, p.938) [48] “Household respondents who were concerned about the safety of the COVID-19 vaccine criticised Hatzola for collaborating with the CVP” (Kasstan et al., 2022, p.2229). [45]
		Empirical articles: [45, 48]. *(n=2)*	
		Program description articles: [27–29, 36, 56]. *(n=5)*	
		Program evaluation articles: [59, 60]. *(n=2)*	
*Category: purpose of partnerships*			
Community-related purpose	Faith health partnerships were sometimes formed to empower and support of faith communities	Theoretical articles: *(n=0)*	“some members were empowered to educate their peers” (Bradley et al., 2018, p.737) [26]
		Empirical articles: *(n=0)*	
		Program description articles: [27, 33–35, 56]. *(n=5)*	
		Program evaluation articles: [26, 64]. *(n=2)*	
Public health purpose	Faith-health partnerships were sometimes used to delivery various public health interventions, including health communication, intervention development, testing, or implementation, or developing partnership or care models.	Theoretical articles: [23, 40]. *(n=2) *	“Respondents valued working collaboratively with faith communities and representatives to deliver information and mental health care so that they can be received positively by targeted communities.” (Abrar & Hargreaves, 2023, p.933) [48] “We sought to partner with faith-based organizations through a community influenza vaccination event to increase vaccination rates.” (Corley et al., 2022, p.1) [55]
		Empirical articles: [28–30, 41, 42, 48, 55]. *(n=7) *	
		Program description articles: [27, 34, 55, 56]. *(n=4) *	
		Program evaluation articles: [26, 37, 59, 60, 64]. *(n=5)*	

### Data items

Extracted data included article characteristics (publication details, country of origin), categorisation of religious group and type of health and wellbeing advocate, intervention details (if relevant), the partnership terminology used, and information about how the partnership was described in the article (Additional File 3, Table 3, Table 4).

**Table 3:** Articles included in scoping review

Author(s)	Year	Article title	Journal title	Article type	Country
Abrar & Hargreaves [48]	2023	Mental health services for Muslim communities in England and Wales: developing a more collaborative model	Mental Health, Religion & Culture	Empirical article	England and Wales
Al-Shaikhali et al. [36]	2023	Providing Free Mammography Screening to Uninsured Muslim Women in South Florida	Journal of Health Care for the Poor and Underserved	Program description article	USA
Bail et al. [60]	2018	Engaging an Urban African American Community to Deliver Cognitive Health Education to Breast Cancer Survivors	Journal of Cancer Education	Program evaluation article	USA
Bateman et al. [56]	2024	Partnering With Churches to Address COVID-19 Vaccine Hesitancy and Uptake in Trustworthy Contexts	American Journal of Public Health	Program description article	USA
Berger [20]	2023	How Can Jewish and Non-Jewish People Collaborate to Improve Healthcare in the US? Considering Community, Autonomy, and Solidarity	Rambam Maimonides Medical Journal	Theoretical article	USA
Bradley et al. [26]	2018	Developing FAITHH: Methods to Develop a Faith-Based HIV Stigma-Reduction Intervention in the Rural South	Health Promotion Practice	Program evaluation article	USA
Bryant [49]	2023	Screening for Social Determinants of Health in Transitional Care Patients and Partnering With the Faith Community to Address Food Insecurity	Professional Case Management	Empirical article	USA
Burt et al. [39]	2024	Faith Community Nursing: Impacting Community-Based Care	Journal of Christian Nursing	Theoretical article	USA
Chaudhary et al. [64][61]	2019	Community Intervention for Syrian Refugees in Baltimore City: The Lay Health Educator Program at a Local Mosque	Journal of Religion and Health	Program evaluation article	USA
Choudhri et al. [17][64]	2024	Cancer-Related Health and Educational Needs and Faith-Based Health Beliefs in an Urban Muslim Population	Journal of Cancer Education	Empirical article	USA
Clements et al. [40]	2021	Using Trauma Informed Principles in Health Communication: Improving Faith/Science/Clinical Collaboration to Address Addiction	Frontiers in Psychology	Theoretical article	USA
Codjoe et al. [44]	2023	Pilot study of a manualised mental health awareness and stigma reduction intervention for Black faith communities in the UK: ON TRAC project	Social Psychiatry and Psychiatric Epidemiology	Empirical article	UK
Corley et al. [55]	2022	Partnering with Faith-Based Organizations to Offer Flu Vaccination and Other Preventative Services	Pediatrics	Program description article	USA
Crankshaw et al. [37]	2020	The Durham Initiative for Stomach Health (DISH): a pilot community-based Helicobacter pylori education and screening study	BMC Gastroenterology	Program evaluation article	USA
El-Majzoub et al. [27]	2021	Negotiating Safety and Wellbeing: The Collaboration Between Faith-Based Communities and Public Health During the COVID-19 Pandemic	Journal of Religion and Health	Program description article	Canada
Epps et al. [63]	2020	Promoting dementia awareness in African-American faith communities	Public Health Nursing	Program evaluation article	USA
Fuller et al. [31]	2024	Reach and Capacity of Black Protestant Health Ministries as Sites of Community-Wide Health Promotion: A Qualitative Social Ecological Model Examination	Journal of Racial and Ethnic Health Disparities	Empirical article	USA
Gore et al. [38]	2022	A Mixed-Methods Formative Evaluation of a Dementia-Friendly Congregation Program for Black Churches	International Journal of Environmental Research and Public Health	Program evaluation article	USA
Gwathmey et al. [33]	2024	Building a Community Partnership for the Development of Health Ministries Within the African American Community: The Triad Pastors Network	Journal of Community Health	Program description article	USA
Hardison-Moody & Yao [57]	2019	Faithful Families, Thriving Communities: Bridging Faith and Health Through a State-Level Partnership	American Journal of Health Promotion	Program description article	USA
Harris et al. [41]	2020	Characteristics Relevant to Respiratory Health Among African Americans Attending Church-based Asthma Programs in Atlanta	Journal of Health Care for the Poor and Underserved	Empirical article	USA
Johs-Artisensi [30]	2021	Faith Community Nursing: A Home-and-Community-Based Partner in Long-Term Care	Journal of Health and Human Services Administration	Empirical article	USA
Kansiewicz & Smith [43]	2021	Implicit and Explicit Impacts of a Church-Based Counseling Program: A Mixed Method Study	Journal of Psychology and Christianity	Empirical article	USA
Kasstan et al. [45]	2022	Localising vaccination services: Qualitative insights on public health and minority group collaborations to co-deliver coronavirus vaccines	Vaccine	Empirical article	England
Kozakowski [23]	2024	Catholic Teaching: A Middle Ground and Guide for End-of-Life Care and Decision-Making and an Antidote for Dying Badly in America	The Linacre Quarterly	Theoretical article	USA
Lynch et al. [42]	2020	Partnering with Churches to Conduct a Wide-Scale Health Screening of an Urban, Segregated Community	Journal of Community Health	Empirical article	USA
Mama et al. [52]	2020	A faith-based mind–body intervention to improve psychosocial well-being among rural adults	Translational Behavioural Medicine	Empirical article	USA
Marin et al. [59]	2019	Adapting Health through Early Awareness and Learning Program into a New Faith-Based Organization Context	Progress in Community Health Partnerships	Program evaluation article	USA
Mattingly et al. [28]	2024	Community-Academic Partnerships for Health Research: An Iterative and Transparent Process of Patient Engagement Before the Research Begins	Ethnicity & Disease	Empirical article	USA
Maxwell et al. [54]	2020	Community health advisors assessing adherence to national cancer screening guidelines among African Americans in South Los Angeles	Preventative Medicine Reports	Empirical article	USA
Maxwell et al. [62]	2019	Promoting Cancer Screening in Partnership With Health Ministries in 9 African American Churches in South Los Angeles: An Implementation Pilot Study	Preventing Chronic Disease	Program evaluation article	USA
Miller [25]	2022	Building trust to cut risk in hard-to-reach groups	Nursing Standard	Theoretical article	UK
Miller [24]	2018	Considering Weight Loss Programs and Public Health Partnerships in American Evangelical Protestant Churches	Journal of Religion and Health	Theoretical article	USA
Milstein & Ferrari [21]	2022	Supporting the wellness of laity: clinicians and Catholic deacons as mental health collaborators	Journal of Spirituality in Mental Health	Theoretical article	USA
Mitchell et al. [50]	2023	University-church partnerships: A mechanism to enhance relationship health	Journal of Prevention & Intervention in the Community	Empirical article	USA
Monson et al. [34]	2021	Congregational COVID-19 Conversations: Utilization of Medical-Religious Partnerships During the SARS-CoV-2 Pandemic	Journal of Religion and Health	Program description article	USA
Moore [35]	2024	Beyond the Hospital Walls: The Role of the Ethicist in Community Healthcare Setting	Journal of Clinical Ethics	Program description article	USA
Olmos-Ochoa et al. [32]	2021	Sustaining Successful Clinical-community Partnerships in Medically Underserved Urban Areas: A Qualitative Case Study	Journal of Community Health Nursing	Empirical article	USA
Parker et al. [53]	2024	Factors Shaping Black Caregivers' Interest and Participation in a University-Church Partnership Program for Youth Mental Health	School Psychology	Empirical article	USA
Peteet et al. [51]	2022	Faith, Fear, and Facts: A COVID-19 Vaccination Hesitancy Intervention for Black Church Congregations	Vaccines	Empirical article	USA
Reed et al. [29]	2024	Breaking chains of tobacco: empowering African American churches in West Virginia for a healthier future	Frontiers in Public Health	Empirical article	USA
Scribner et al. [58]	2020	Bridges to Care and Recovery: Addressing Behavioral Health and Mental Health Needs Through the Faith Community	Journal of Religion and Health	Program description article	USA
Williams et al. [47]	2023	Lessons Learned about Developing Faith and Public Health Partnerships to Address Health Disparities	Community Health Equity Research & Policy	Empirical article	USA
Williams et al. [22]	2021	Combating Contagion and Injustice: The Shared Work for Public Health and Faith Communities During COVID-19	Journal of Religion and Health	Theoretical article	USA
Zimmermann et al. [46]	2023	Application of the consolidated framework for implementation research to understand implementation context of a cardiovascular disease risk-reduction intervention in rural churches	Translational Behavioral Medicine	Empirical article	USA

**Table 4:** Partner and intervention details for articles included in scoping review

Authors (Year)	Methodology	Methods	Partner details: Faith community	Partner details: health & wellbeing advocate	Health need targeted by intervention	Target population	Intervention	Intervention settings
Abrar & Hargreaves (2023) [48]	Qualitative	Focus group	Muslim	Clinical; Allied health	Increase cultural appropriateness of mental health services	Muslim communities	Mental health services	Community
Al-Shaikhali et al. (2023) [36]	Quantitative	Survey	Muslim	Academic; Clinical	Increase in uptake of mammography screening	Uninsured Muslim Women	Education program; cancer screening	Place of worship; Online
Bail et al. (2018) [60]	Quantitative	Survey, demographic questionnaire	Christian	Academic	Improving cognitive function in breast cancer survivors	African Americans	Education program	Place of worship
Bateman et al. (2024) [56]	Qualitative	Survey, focus groups and interviews	Christian	Academic; Public health	Increase uptake of COVID-19 vaccine	Black communities	Education program; mobile vaccination clinics	Place of worship
Berger (2023) [20]	Not relevant	N/A	(Jewish)	Other: (Theoretical partnerships)	No intervention	(Jewish and non-Jewish Americans)	(Restructuring health institutions) (proposed)	(Not specified)
Bradley et al. (2018) [26]	Mixed methods	Interviews, survey	Christian	Academic; Community organisation	Reduce stigma about HIV/AIDS; reduce poor wellbeing in people living with HIV	African American communities	Education program	Place of worship
Bryant (2023) [49]	Qualitative	Survey; questionnaire	Christian	Clinical	Address Social Determinants of Health (SDOH) and food insecurity	Patients experience food insecurity	SDOH screening; provision of nonperishable food; distribution of service information	Other: medical clinic
Burt et al. (2024) [39]	Not relevant	N/A	(Christian)	Other: (theoretical partnership—faith community nursing)	(Health and wellbeing generally)	(Church attendees)	(Faith community nursing)	(Community; Place of worship)
Chaudhary et al. (2019) [61]	Quantitative	Survey	Muslim	Other: University hospital	Increase health literacy	Refugees, predominantly Muslim	Peer education program	Community; Place of worship
Choudhri et al. (2024) [64]	Quantitative	Survey	Muslim	Academic	Increase knowledge of cancer-related needs for Muslims	Muslim communities	Screening for cancer-related health needs	Place of worship; Online
Clements et al. (2021) [40]	Not specified	N/A	(Christian)	(Academic; Public health; Clinical; Other: Scientific)	(Addiction)	(Church attendees)	(Collaboration to improve health communication)	(Place of worship)
Codjoe et al. (2023) [44]	Mixed methods	Scales; surveys; interviews; focus group	Christian	Other: mental health services	Reduce stigma about mental health	Black Majority Church (BMC) attendees	Education program	Community; Online
Corley et al. (2022) [55]	Quantitative	survey	Christian	Clinical; Other: Faith-affiliated health advocacy organisation	Increase paediatric influenza vaccination rates and increase vaccine literacy	Children and families; Black/African American communities	Vaccination and education programs	Place of worship
Crankshaw et al. (2020) [37]	Quantitative	survey, specimen collection	Christian	Academic; Clinical	Increase h. pylori testing and eradication	African American communities	Health screening; education program	Place of worship
El-Majzoub et al. (2021) [27]	Qualitative	Case study	Muslim; Jewish	Public health; Clinical; Allied health; Other: Police	Increase compliance to COVID-19 restrictions	Jewish and Muslim communities	Negotiation with faith communities	Online
Epps et al. (2020) [63]	Quantitative	Surveys	Christian	Coalition/network	Dementia awareness	African American congregations	Education program	Place of worship
Fuller et al. (2024) [31]	Qualitative	Interviews	Christian	Academic; Other: community advisory board	Health and wellbeing generally	Black Protestant congregations	Public health partnership with Black churches for education programs	Community; Place of worship
Gore et al. (2022) [38]	Mixed methods	Survey; focus groups	Christian	Academic; Community organisation	Dementia awareness and support	Predominantly African American congregations	Education and support program	Place of worship
Gwathmey et al. (2024) [33]	Mixed methods	Survey; focus group	Christian	Academic	Improve health equity and reduce health disparities	African American communities	Partnership with African American communities for health ministry resourcing	Place of worship
Hardison-Moody & Yao (2019) [57]	Mixed methods	Survey, focus group	Christian	Academic; Public health	Improve diet and increase physical activity	Minority and low-income communities	Education program	Community; Place of worship
Harris et al. (2020) [41]	Quantitative	survey, spirometry	Christian	Clinical	Improve asthma outcomes	African American communities	Education and advocacy program	Place of worship
Johs-Artisensi (2021) [30]	Quantitative	surveys	Christian	Clinical	Health and wellbeing generally	Older Americans	Faith Community Nursing	Community; Place of worship
Kansiewicz & Smith (2021) [43]	Mixed methods	Survey; case study	Christian	Academic	Increase access to mental health treatment and reduce mental health stigma	Christian congregations	Clinical mental health counseling	Place of worship
Kasstan et al. (2022) [45]	Qualitative	Interviews	Jewish	Public health	Increase uptake of COVID-19 vaccination	Haredi Jewish communities	Vaccination program	Community
Kozakowski (2024) [23]	Not relevant	N/A	(Christian)	Other: (theoretical partnership)	(End-of-life care)	(Catholic communities)	(Collaboration between clinical and religious communities for end-of-life care)	(Place of worship; Other: clinical)
Lynch et al. (2020) [42]	Quantitative	Physical health assessments, questionnaires	Christian	Academic	Health equity	African Americans	Health screening program	Place of worship
Mama et al. (2020) [52]	Quantitative	Physical health assessments, questionnaires	Christian	Academic; Allied health	Increase physical actively and reduce psychological distress	Rural adults who were overweight or obese	Physical activity and relaxation program	Place of worship; Other: university
Marin et al. (2019) [59]	Quantitative	Survey	Christian	Academic; Clinical; Other: Health care chaplain	Health and wellbeing generally	Underserved communities	Community Health Advisor and Education Program	Place of worship
Mattingly et al. (2024) [28]	Qualitative	workshop	Christian	Academic; Community organisation	Increasing COVID-19 testing	African American communities	Model for Academic-Community partnership to mitigate disparities and inequities	Community; Place of worship
Maxwell et al. (2019) [62]	Quantitative	Survey	Christian	Academic	Increase cancer screening adherence	African American communities	Adherence assessment and education program delivered by Community Health Advisors	Place of worship; Phone
Maxwell et al. (2020) [54]	Quantitative	Survey	Christian	Academic	Increase cancer screening adherence	African American communities	Adherence assessment and education program delivered by Community Health Advisors	Community; Place of worship; Phone
Miller (2018) [24]	Not relevant	N/A	(Christian)	Other: (Theoretical partnership—public health)	(Obesity)	(American Evangelical Protestant (AEP) Church attendees)	(Collaboration between public health and AEP weight loss programs.)	(Community; Place of worship)
Miller (2022) [25]	Not specified	Not specified	(Muslim; Jewish; Sikh; Faith communities generally; Other: Faith leaders in Travelling community)	(Academic; Clinical)	(Multiple. Increase in vaccine uptake and stem cell donation, cancer screening, cardiac health, organ donation)	(Multiple. Sikh community; traveller community; Muslim community; some ethnic communities)	(Health promotion events, health screening, cancer screening, education program.)	(Community)
Milstein & Ferrari (2022) [21]	Mixed methods	Survey	(Christian)	Other: (Theoretical – clinical)	(Improve the continuity of mental health care)	(Catholic communities)	(Model for collaboration between mental health services and faith communities.)	(Community)
Mitchell et al. (2023) [50]	Mixed methods	Survey	Christian	Academic	Reducing relationship distress	Church attending couples	Education program delivered by trained lay people	Place of worship
Monson et al. (2021) [34]	Not specified	N/A	Christian; Muslim; Jewish	Other: university hospital	Health messaging and COVID-19 mitigation	Christian, Jewish and Muslim faith communities	Health messaging	Online
Moore (2024) [35]	Not specified	N/A	Christian	Academic; Clinical; Allied health	Increase access to health services	Underserved communities	Partnership to deliver community clinic providing health services	Place of worship
Olmos-Ochoa et al. (2021) [32]	Qualitative	case study; interviews	Christian	Community organisation	Improve delivery of preventive health services outside the health system	Underserved communities	Partnership to deliver preventative health screening	Place of worship
Parker et al. (2024) [53]	Qualitative	interview	Christian	Academic	Child and youth mental health	Black K-12 school students'	Virtual Mentoring	Online
Peteet et al.(2022) [51]	Quantitative	survey	Christian	Academic; Public health; Community organisation	Reduce medical mistrust and increase uptake of COVID-19 vaccination	Black church congregations	Partnership for education program	Online
Reed et al. (2024) [29]	Quantitative	Survey	Christian	Community organisation; Coalition/network	Increase cessation rates of tobacco use; tobacco prevention	African Americans in West Virginia	Education program delivered through trained lay leaders	Community; Place of worship
Scribner et al. (2020) [58]	Mixed methods	survey, focus groups, interviews	Christian	Public health; Clinical; Community organisation	Increase access to mental health services and reduce stigma about mental health	African American communities	Training lay leaders as "Wellness Champions"	Community; Place of worship
Williams et al. (2023) [47]	Qualitative	Interviews	Christian	Academic; Public health	Obesity and diabetes prevention	African American and Latino congregations	Partnership to deliver interventions	Community
Williams et al. (2021) [22]	Not relevant	N/A	(Faith communities generally)	Other: (theoretical—academic, public heath, community organisation)	(Communities flourishing; prevention of vaccine preventable diseases)	(Faith communities)	(Collaborative dialogue)	(Not specified)
Zimmermann et al. (2023) [46]	Qualitative	Interviews; case study	Christian	Other: University hospital	Improve dietary and physical activity behaviours to reduce CVD risk	Rural women	Dietary and physical activity program	Place of worship

Information related to theoretical partnerships have been enclosed in round brackets to distinguish them from empirical partnerships

## Results

### Selection of sources of evidence

In total, 1018 articles were screened for relevance to the review concepts. Screening of titles and abstracts resulted in the exclusion of 817 articles. A further 156 articles were excluded following full text screening. Reasons for exclusion were: interventions delivered through CBPR partnerships, or lack of any health or wellbeing intervention (wrong concept, *n* = 40), lack of a faith-health partnership or collaboration (wrong concept, *n* = 53) wrong type of article (*n* = 29) lack of focus on faith communities (*n* = 17), wrong country (*n* = 15), no local intervention (*n* = 1), and no health and wellbeing partner (*n* = 1). A total of 45 articles were included in the final review (Fig. 1).

### Characteristics of sources of evidence

#### Country of origin

Most articles included in the review were from the USA (*n* = 40), two were from the UK, one from both England and Wales, one from England only and one from Canada. (Table 3).

### Type of article

Most articles were primary research articles (*n* = 37), which were categorised into empirical articles (*n* = 20), program descriptions (*n* = 9) and program evaluations (*n* = 8). Empirical articles examined the effects of an intervention. Program descriptions articulated the development of partnerships or interventions and may or may not have included demographic statistics. Program evaluations examined programs in terms of their acceptability and feasibility. The review also included eight theoretical articles about the potential benefits of faith-health partnerships. (Table 3).

### Aims of articles

The aims of the articles included were categorised into those which were collaboration-focused, and those which were intervention-focused. Collaboration-focused aims were identified in all article types: theoretical articles (*n* = 6), empirical articles (*n* = 5), program descriptions (*n* = 5) and program evaluations (*n* = 3). Collaboration-focused aims included describing collaboration (*n* = 8), discussing theoretical aspects of partnership (*n* = 6), describing models of partnerships (*n* = 2), or evaluating potential partners (*n* = 2). Similarly, intervention-focused aims were identified in all article types; theoretical articles (*n* = 3), empirical articles (*n* = 15), program descriptions (*n* = 5) and program evaluations (*n* = 7). Intervention-focused aims involved describing a program or intervention (*n* = 7), evaluating an intervention (*n* = 7)**,** reporting on a pilot intervention (*n* = 4), describing intervention development (*n* = 4), exploring acceptability of interventions with specific populations (*n* = 2), reporting key findings from faith-health partnerships (*n* = 3), identifying health needs for specific populations (*n* = 3), and examining the impact of context on an intervention (*n* = 1).

### Methodology and methods for primary research articles

Methodology for the included articles was identified as either quantitative (*n* = 16), qualitative (*n* = 11), mixed methods (*n* = 9) or not relevant/no methods. Theoretical articles did not specify methods (*n* = 7) or used mixed methods (*n* = 1). Empirical articles used qualitative methods (*n* = 9), quantitative methods (*n* = 8), and mixed methods (*n* = 3). Program descriptions used qualitative (*n* = 2), quantitative (*n* = 2) or mixed methods (*n* = 3), or did not specify methods (*n* = 2). Program evaluations used quantitative (*n* = 6) and mixed methods (*n* = 2).

Quantitative methods used in the articles included surveys (*n* = 25), questionnaires (*n* = 4), scales (*n* = 1), and other forms of health screening data, for example, spirometry, specimen or physical health assessment measures (*n* = 5). Qualitative methods included interviews (*n* = 9), focus groups (*n* = 7), case studies (*n* = 4), and workshops (*n* = 1) (Table 3).

### Partner: faith community

Partner characteristics were divided into theoretical partnerships, which feature in theoretical articles, and empirical partnerships, which feature in primary research articles. Christian faith communities featured most prominently as both empirical (*n* = 32) and theoretical partners (*n* = 4). Other theoretical partners were Jewish (*n* = 1) and faith communities generally (*n* = 1). Empirical partners from non-Christian faith communities were Muslim (*n* = 7), Jewish (*n* = 4), and Sikh (*n* = 1) faith communities, as well as faith leaders in Traveller communities (*n* = 1), or faith communities generally (*n* = 1) (See Table 4).

### Partner: health and wellbeing advocate

The health and wellbeing partners that featured in the articles were from the following fields or professions: academic (*n* = 22), clinical (*n* = 11), public health (*n* = 7), community organisations (*n* = 8), allied health (*n* = 4), coalition/network (*n* = 2) and other, which included, university hospitals (*n* = 3), mental health services (*n* = 1), police (*n* = 1), community advisory board (*n* = 1), a health care chaplain (*n* = 1), scientific (*n* = 1), and a faith-affiliated health advocacy organisation (*n* = 1). Health and wellbeing partners who were discussed as theoretical partners included academics (*n* = 3), clinical (*n* = 1), public health (*n* = 3), community organisations (*n* = 1), and scientific (*n* = 1) partners (Table 4).

### Health need addressed by partnership

A diverse range of health issues was targeted by the interventions delivered through the faith-health partnerships described in the primary research literature, including COVID-19 (*n* = 6), mental health (*n* = 5), cancer-related needs (*n* = 5), metabolic diseases such as obesity and diabetes (*n* = 3), general health and wellbeing (*n* = 3), dementia (*n* = 2), substance use/addiction (*n* = 1), asthma (*n* = 1), relationship distress (*n* = 1), influenza (*n* = 1), h-pylori infection (*n* = 1), health literacy (*n* = 1), and human immunodeficiency virus (HIV) (*n* = 1). Partnerships were also a key strategy used to try and reduce health inequalities (*n* = 4). Theoretical articles described the potential contribution of faith-health partnerships to health needs such as general health and wellbeing (*n* = 2), vaccine preventable diseases (*n* = 2), obesity (*n* = 1), mental health care (*n* = 1), end-of-life care (*n* = 1), cardiovascular disease (*n* = 1), and cancer screening (*n* = 1) (Table 4).

### Interventions featured

Interventions delivered through faith-health partnerships included health education programs (*n* = 14), lay or peer education programs (*n* = 6), health screening (*n* = 4), vaccination programs (*n* = 3), mental health services (*n* = 2), faith community nursing programs (*n* = 1), physical activity programs (*n* = 2), cancer screening (*n* = 2), screening for social determinants of health (*n* = 1), screening for cancer-related needs (*n* = 1), provision of food packages (*n* = 1), health messaging (*n* = 1), health services (*n* = 1), and mentoring (*n* = 1). In many cases, collaboration was identified as a key element of intervention (*n* = 13). Theoretical articles included descriptions of potential interventions including collaboration as an intervention strategy (*n* = 5), faith community nursing (*n* = 1), and a proposed restructure of health institutions (*n* = 1) (Table 4).

### Target population

Many interventions provided through faith-health partnerships targeted minority ethnic or religious communities such as African American/Black communities (*n* = 18); Muslim communities (*n* = 6), and Jewish communities (*n* = 3). Other faith-health partnership interventions aimed to meet the needs of “underserved” communities (*n* = 3), rural adults (*n* = 2) or those experiencing other forms of health inequalities (*n* = 3). Some interventions sought to target those were not specifically minorities but were church attendees (*n* = 3), older adults (*n* = 1), or children (*n* = 1). Theoretical articles proposed that faith-health partnerships could be used to help reach target populations such as church attendees (*n* = 5), faith communities generally, (*n* = 1), Muslim communities (1), Jewish communities (*n* = 1), Sikh communities (*n* = 1), and traveller communities (*n* = 1). (Table 4).

### Intervention setting

A range of intervention settings was featured in the primary research, including places of worship (*n* = 28), community settings (*n* = 12), online platforms (*n* = 7), and over the phone (*n* = 2). At times the intervention setting was not specified (*n* = 1). Other settings of interventions included a medical clinic (*n* = 1) and a university research centre (*n* = 1). The settings suggested in the theoretical articles for faith-health partnership delivered interventions included community (*n* = 4), places of worship (*n* = 4), clinical settings (*n* = 1), or did not specify a location (*n* = 1) (Table 4).

### Results of individual sources of evidence

See Table 3 and 4

### Synthesis of results

#### Terminology

The main terms used to describe the collaborative relationship between faith communities and health and wellbeing organisations and professionals in the literature were variations of the term partner/partnership (*n* = 44), collaborate/collaboration/collaborative (*n* = 29), engage/engaging/engagement (*n* = 7), joint working/working together/working with (*n* = 5). Other terms identified that implied a level of cooperative working included network (*n* = 2), co-design (*n* = 1), co-deliver (*n* = 1), co-creation (*n* = 1), co-led (*n* = 1).

#### How is partnership described in the literature?

Theoretical articles explored the potential contribution of faith-health partnerships by examining the history of contribution from particular faith communities, for example, the Jewish community [20] or Catholic deacons [21], or the history of development of faith community nursing [39]. Other theoretical articles proposed faith-health partnerships as a means of addressing health issues like COVID-19 [22] or end-of-life care [23], or the implications of, or for, faith-health collaboration for specific interventions or programs [24, 65].

#### Purpose of partnership

In the primary research articles, faith-health partnerships were often described in relation to their purpose. Partnerships were used as a strategy to develop [26, 28, 29, 37, 41, 42, 59, 64], deliver [20, 30, 38, 41, 43, 55, 60] or test [26, 44, 56] a range of public health interventions. Partnerships were also used to provide support to faith communities [33], including advice on how to reopen faith communities during the COVID-19 pandemic [27, 34], or training, technical support [56], or resources [61]. Some partnerships spanned several levels, from the local to the state [57] or national level [29], and some included multiple levels of involvement for faith communities [33].

#### Challenges

Challenges in partnerships were noted in many of the primary research articles, but rarely in the theoretical literature, with the exception of one theoretical article which discussed the potential difficulty presented by partnering with faith communities who were implementing programs based on theological positions that conflicted with public health perspectives [24]. Challenges were mentioned in empirical articles (*n* = 3), program descriptions (*n* = 4) and a program evaluation (*n* = 1). Challenges were broadly categorised as intervention-related challenges and relational challenges. Intervention-related challenges included difficulties with recruitment, buy in and logistics [26], partner readiness, lack of fidelity to the interventions and challenges with data collection [57]. A lack of long-term funding was also identified as a challenge [45]. Relational challenges included differences between partners, including values [24, 35] and priorities [24, 46]. Stigma, distrust and a lack of knowledge about faith communities were also challenges [47], along with negative responses to potential partnership from within faith or cultural communities [56].

#### Facilitators

Facilitators to partnership were also noted in the literature. Both theoretical (*n* = 5) and primary research articles (empirical: *n* = 18, program descriptions: *n* = 9, program evaluations: *n* = 8) reported facilitators of their interventions and partnerships. These included asset-related facilitators, and relational facilitators; often these overlapped. Theoretical articles proposed that successful partnership was enhanced by existing assets or infrastructure in the faith community [22, 39], and recognition of the expertise and knowledge of each partner [22, 23]. These facilitators were both noted in the primary research articles, with expertise and knowledge assisting collaborative efforts, including having previous involvement in health-related interventions [36, 48, 56, 60]. Sharing assets, such as buildings and volunteers, was frequently cited as a common behaviour in faith-health partnerships in the primary research articles [27, 30–33, 45, 49–51, 56–58]. Building the capacity of faith communities for public health related work was also important in maintaining partnerships [47, 57, 64].

Additional facilitation noted in the primary research articles included pre-existing relationships and networks providing avenues through which partnerships could be built [33, 34, 36, 37, 42, 45, 49, 51, 52, 55, 62]. Building trust with faith partners and their wider communities was identified as a key facilitator in developing and maintaining the partnerships [25, 26, 28, 33, 37, 47, 53, 55, 58, 63]. As such, the reputation of collaborating organisations was important in establishing partnerships. Having reputable partners involved increased community trust in the intervention [53], and gave validity to partnerships [29]. Trusted leaders, both from within faith communities, and in health and wellbeing organisations, were identified as key assets in faith-health partnerships in both the theoretical [21, 25] and primary research articles, [26, 27, 29, 34, 41, 45, 46, 48, 51, 54, 55, 57, 59, 60, 62], and their presence could increase trust in intervention settings [37, 45]. Few articles included detail about how trusted leaders were identified, however it was implied that people within the faith communities or within a given profession may be best placed to identify who is considered credible and influential from within their own spheres [29, 34, 48]. Related to the need for trusted leaders is the recognition of the importance of people who may be considered “bridge-builders” [40, p.7]. Theoretical articles described bridge-building being facilitated by determining when to draw on the expertise of other professions [21], and networking between health professionals and faith communities [39]. Recognition of the expertise of health and faith partners was affirmed as beneficial to collaboration in the primary research articles [33, 47, 48, 57] and those with experience in health care or academic settings as well as in faith communities drew on their expertise to build these bridges [28, 31, 48, 59]. Bridge-builders could also facilitate dialogue around areas of conflict [35].

Other facilitators noted throughout the primary research evidence included flexibility when setting up faith-heath partnerships [26, 32, 47, 58], common aims and understandings [30, 32, 35, 47, 52], and creating buy-in with communities [26] which may help build accountability for intervention outcomes [29].

#### Benefits of the partnership.

This review has not examined the population health and wellbeing outcomes resulting from the partnerships interventions explored in the literature. Many of the articles did not provide this type of data, as their focus was on theoretical discussion of faith-health partnerships, or on describing or measuring results of interventions, rather than collaboration. However, some of the benefits of partnerships were noted during extraction in the primary research articles. Involvement in faith-health partnerships was good for organisational reputation [35]. Health partners came to recognise that faith partners shared common aims to improve the health and wellbeing of their communities [27]. Increased community trust in partners and interventions was also recognised as a benefit of faith-health partnerships [28, 53], although this was not always the case [45]. Partnerships of this kind helped ensure interventions were culturally relevant, and some led to the development of other faith-health projects or initiatives [27, 60].

## Discussion

### Summary of evidence

The question guiding this scoping was: What is known from the current literature about the partnerships between faith communities and health and wellbeing advocates who work together to improve the health and wellbeing of the local communities? The reviewed literature indicated that faith-health partnerships feature in a heterogeneous range of academic literature, including primary research articles and theoretical articles. In the reviewed articles, faith-health partnerships were used to address several health and wellbeing needs through a range of interventions. However, much of this research in the selected countries has been conducted in the USA. There appears to be a paucity of literature examining faith-health partnerships in the other countries. Future research could focus on how faith-health partnerships work in other high-income countries that are similar to the USA in terms of culture, and diversity of population. The included articles frequently used variations of terms such as partnership and collaboration, but these terms were rarely defined. Rather, authors tended to provide descriptions of their partnerships and/or intervention processes, at times only minimally. This may be because many of the reviewed articles were intervention-focused, especially empirical articles. Collaboration or partnership was usually mentioned in these articles when describing the development or delivery of interventions, or where intervention delivery or outcomes were impacted by how partners worked together. In contrast, discussion about collaboration in faith-health partnerships was often the focus of theoretical articles. This is expected, given that theoretical articles were not concerned with application of a program or intervention. Many articles included articulations of the challenges and facilitators related to their partnerships and interventions. The extracted data included many more facilitators to partnership, than challenges. However, frequency counts should not be read to assume that there are more benefits to partnership than there are challenges, only that perhaps authors are more likely to report facilitators. It was not unusual for facilitators and challenges to be listed or shortly described, rather than explained. Therefore, more research is needed to understand the mechanisms that cause these factors to act as barriers and/or facilitators to collaboration. In addition, research is needed to determine whether faith-health partnership delivered interventions create significant and lasting change in health and wellbeing outcomes for the target populations.

### Limitations

This review has some limitations. As the data extracted were conceptual in nature, it was difficult to develop an extraction form that could accurately capture the heterogeneous nature of the data included in the articles. It also required judgement on behalf of the reviewers who were screening and extracting the data. This was managed by ensuring all screening and data extraction involved two or more reviewers. Most of the research included in this review came from the USA. This may be due to the limited number of databases included. For example, Scopus and Web of Science were not used to source literature for the review. The exclusion of these databases may have limited the comprehensiveness of this review. In addition, as the review team consisted of researchers from Australia and the UK, it is possible that some of the conceptual categories developed for the review do not align with how partnerships are broadly understood to operate in the USA. However, this also increased the likelihood that conceptual categories used in the review were relevant across both the UK and Australia, and to the settings of the research which the scoping review informed. Furthermore, this review excluded literature that featured CBPR partnerships, faith-heath partnerships in other parts of the world, international faith-health partnerships, and articles written in languages other than English. A review of the literature which includes these approaches and partnerships in other contexts may yield different results to those found here. Finally, this review did not include grey literature. It is possible that faith-health partnerships which do not involve academics participating in the project function and report their activities differently.

## Conclusion

Academics, health and wellbeing advocates, and faith leaders who are looking to establish faith-health partnerships could learn from the challenges and facilitators described in this review. More research is needed to understand the full scope of faith-health partnerships beyond the academic literature and beyond the USA, and to determine their impacts on community and population health and wellbeing.

## Supplementary Information

Additional file 1. Flow chart for Scoping Review Inclusion and Exclusion.

Additional file 2. Screenshots of data extraction form for Scoping Review on faith-health partnerships.

Additional file 3. Extracted data.

## Data Availability

All data generated or analysed during this study are included in this published article [and its supplementary information files].

## Abbreviations

CBPR: Community-Based Participatory Research
HIV: Human immunodeficiency virus
PCC: Population Concept Context
UK: United Kingdom
USA: United States of America
