# Supplementary material for: Sketching the landscape: a scoping review of partnerships at the intersection of faith and health
Source: BMC Public Health. 2025 Dec 19;25:4266. doi: 10.1186/s12889-025-25346-9 (PMC12717692; doi:10.1186/s12889-025-25346-9)
Supplement: Supplementary file 1 — Additional file 1. Flow chart for Scoping Review Inclusion and Exclusion. [file 12889_2025_25346_MOESM1_ESM.docx]

**Flow chart for Scoping Review Inclusion and Exclusion**

E.g. Exclude international projects

*Local not needed for theoretical papers

E.g. Exclude if not specific to faith communities. I.e. they’re one of many community partners

YES

YES

YES

YES

YES

YES

NO

NO

NO

NO

NO

NO

NO

E.g. Exclude news articles, newsletters, theses.

Include in review: theoretical papers

Exclude from review: CBPR at this level

E.g. Exclude if faith community are participants only

Exclude as wrong type of paper

Exclude as wrong context:

Wrong country

Exclude as wrong context:

Not local

Exclude as wrong concept: no partnership or collaboration

Exclude as wrong concept: no or wrong health intervention

Exclude as wrong population: no health advocate

Exclude as wrong population: no faith community focus

Literature type:

Peer reviewed journal articles.

Context 1:

Latin Western countries, excluding Latin America and the Orthodox World. (include Western Europe and UK, Iceland Scandinavia, USA, Canada, Australia, NZ)

Context 1:

Local intervention

Concept 2:

Health and wellbeing intervention or need

Concept 1:

Partnership or collaboration

Population 2:

Health and wellbeing advocates

Population 1:

Faith communities
