# Supplementary material for: Sketching the landscape: a scoping review of partnerships at the intersection of faith and health
Source: BMC Public Health. 2025 Dec 19;25:4266. doi: 10.1186/s12889-025-25346-9 (PMC12717692; doi:10.1186/s12889-025-25346-9)
Supplement: Supplementary file 2 — Additional file 2. Screenshots of data extraction form for Scoping Review on faith-health partnerships. [file 12889_2025_25346_MOESM2_ESM.docx]

**Additional file 3: Screenshots of data extraction form for scoping review on faith-health partnerships
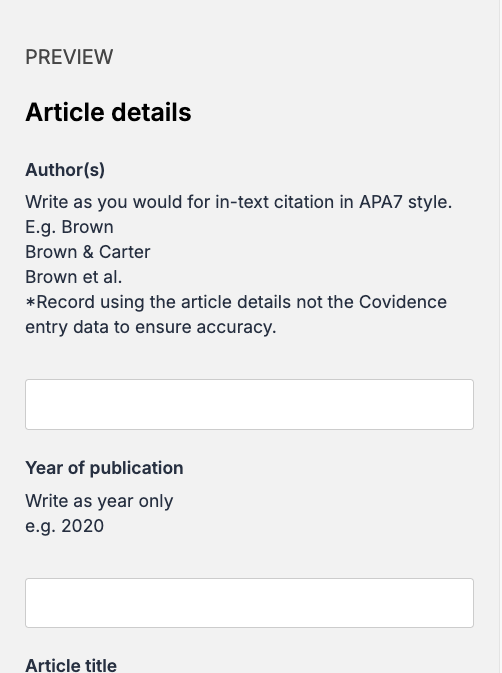
**

**
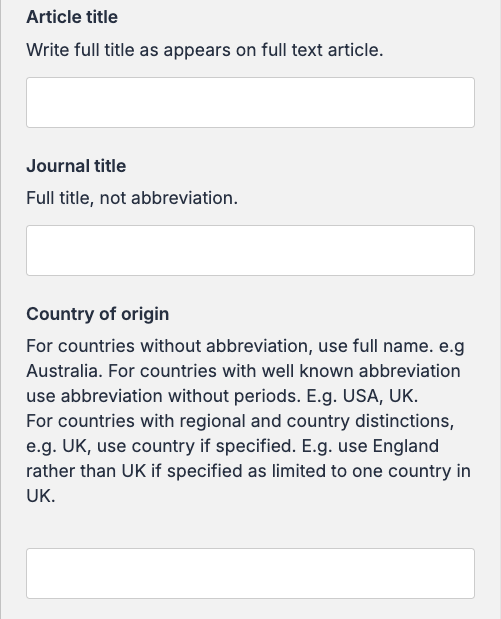

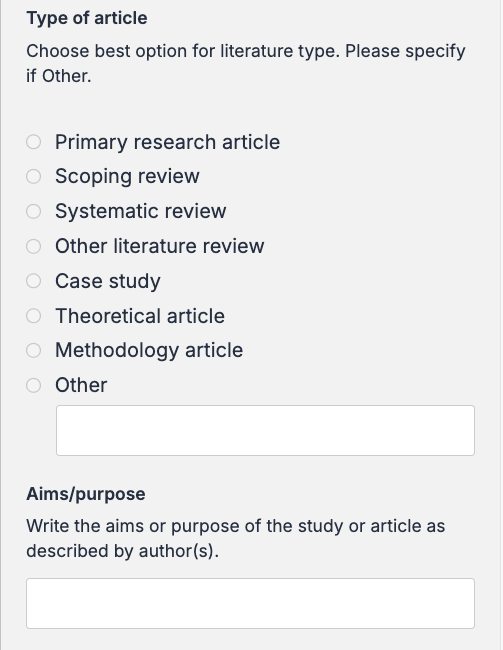

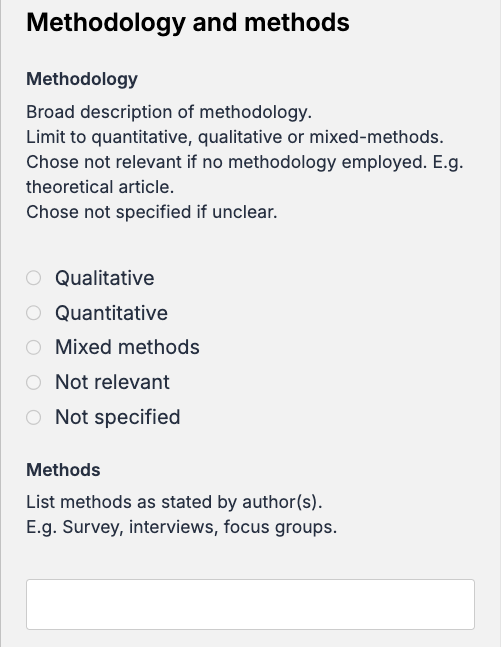

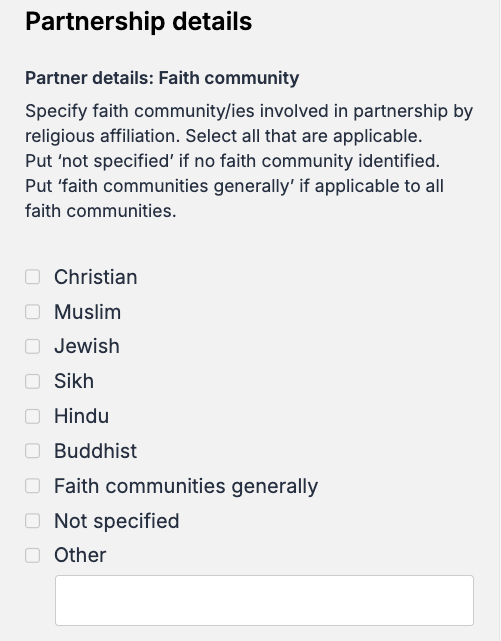

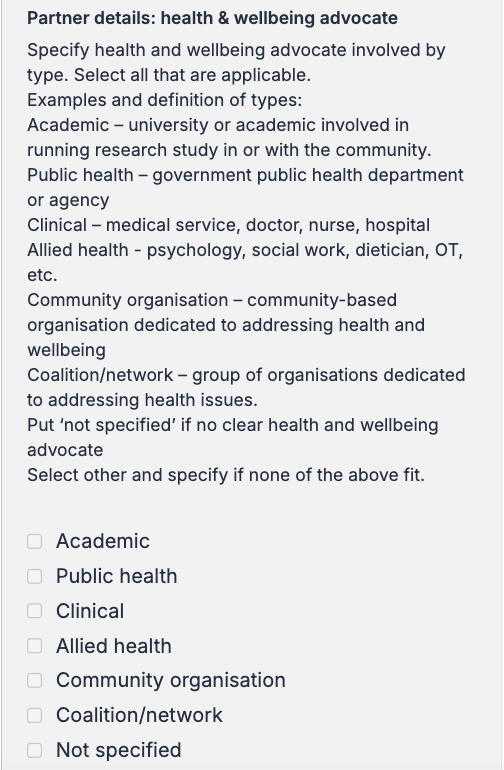

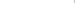

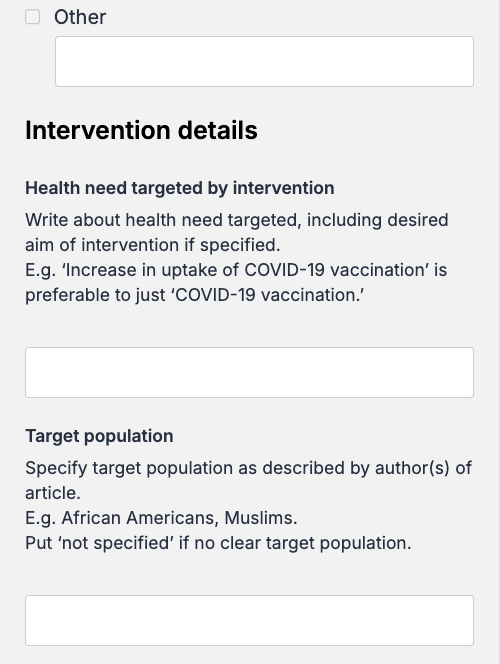

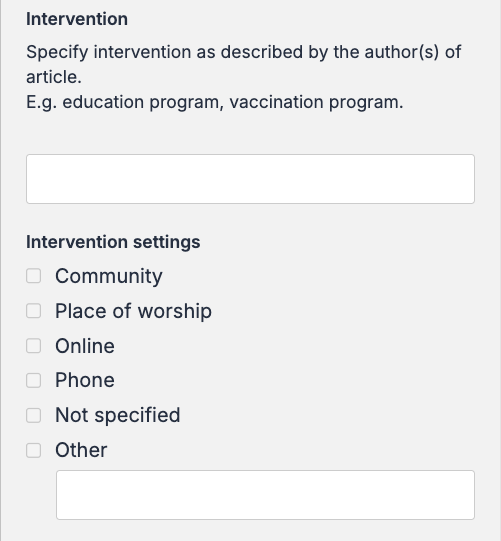

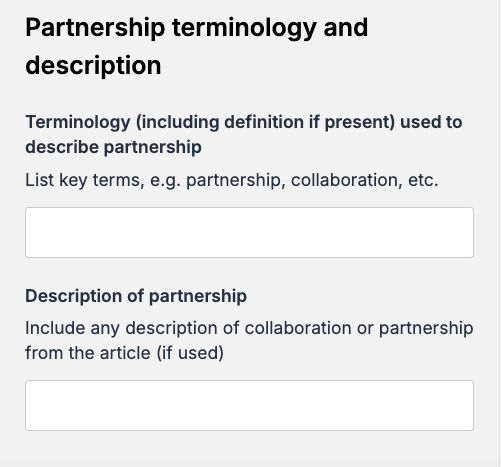
**
